# Incidence of obesity during childhood and adolescence in a large contemporary cohort^☆^

**DOI:** 10.1016/j.ypmed.2011.02.014

**Published:** 2011-05-01

**Authors:** Adrienne R. Hughes, Andrea Sherriff, Debbie A. Lawlor, Andrew R. Ness, John J. Reilly

**Affiliations:** a University of Stirling, Department of Sports Studies, Stirling, FK9 4LA, Scotland, UK; b University of Glasgow Dental School, 378 Sauchiehall Street, Glasgow, G2 3JZ, Scotland, UK; c MRC Centre for Causal Analyses in Translational Epidemiology, Department of Medicine, Oakfield House, Oakfield Grove, Clifton, Bristol, BS8 2BN, UK; d University of Bristol, Department of Oral and Dental Science, Oakfield House, Oakfield Grove, Clifton, Bristol, BS8 2BN, England, UK; e Physical Activity for Health Research Group, University of Strathclyde School of Psychological Sciences and Health, Jordanhill Campus, 76 Southbrae Drive, Glasgow, G13 1PP, Scotland, UK

**Keywords:** ALSPAC, Obesity, Aetiology, Prevention, Overweight, Children, Adolescents

## Abstract

**Background and Aims:**

Timing of obesity development during childhood and adolescence is unclear, hindering preventive strategies. The primary aim of the present study was to quantify the incidence of overweight and obesity throughout childhood and adolescence in a large contemporary cohort of English children (the Avon Longitudinal Study of Parents and Children, ALSPAC; children born 1991–1992). A secondary aim was to examine the persistence of overweight and obesity.

**Methods:**

Longitudinal data on weight and height were collected annually from age 7–15 years in the entire ALSPAC cohort (n = 4283), and from 3 to 15 years in a randomly selected subsample of the cohort (n = 549; ‘Children in Focus’ CiF). Incidence of overweight and obesity (BMI (Body mass index) at or above the 85th and 95th centiles relative to UK reference data) was calculated. Risk ratios (RR) for overweight and obesity at 15 years based on weight status at 3, 7, and 11 years were also calculated.

**Results:**

In the entire cohort, four-year incidence of obesity was higher between ages 7 and 11 years than between 11 and 15 years (5.0% vs 1.4% respectively). In the CiF sub-sample, four-year incidence of obesity was also highest during mid-childhood (age 7–11 years, 6.7%), slightly lower during early childhood (3–7 years, 5.1%) and lowest during adolescence (11–15 years 1.6%). Overweight and obesity at all ages had a strong tendency to persist to age 15 years as indicated by risk ratios (95% CI (Confidence interval)) for overweight and obesity at 15 years from overweight and obesity (relative to healthy weight status) at 3 years (2.4, 1.8–3.1), 7 years (4.6, 3.6–5.8), and 11 years (9.3, 6.5–13.2).

**Conclusion:**

Mid–late childhood (around age 7–11 years) may merit greater attention in future obesity prevention interventions.

## Introduction

Prevalence of childhood obesity rose markedly in the UK from the late 1980s (Reilly and Dorosty, 1999, Reilly et al., 1999, McCarthy et al., 2003). Recent UK trends suggest that the rate of increase in obesity prevalence may have slowed (Stamatakis et al., 2010), as in some other countries (Han et al., 2010). However, social patterning of overweight and obesity in UK children and adolescents is increasing (Stamatakis et al., 2010).

Many studies of obesity *prevalence* have taken place, but there is a dearth of evidence on the ‘natural history’ of obesity (Whitaker, 2002, Reilly et al., 2007). Only a few studies have reported on the *incidence* of child and adolescent obesity (Andersen et al., 2010, Gortmaker et al., 1996, Hesketh et al., 2003, Nader et al., 2006, Plachta-Danielzik et al., 2010), and none have reported on incidence across childhood and adolescence. Evidence on incidence of overweight and obesity by age group would be helpful to prevention strategies: periods of highest incidence might merit highest priority in preventive interventions. A recent review (Nichols and Swinburn, 2010) found that decision-making in choice of target population for obesity prevention is rarely explicit. Specific periods of childhood and adolescence might be particularly important to the establishment of health behaviours related to obesity, and identifying whether incidence of obesity is highest in early childhood (e.g. 3–7 years), mid–late childhood (7–11 years), or adolescence (beyond 11 years) could inform preventive interventions.

The primary aim of the present study was therefore to estimate the incidence of overweight and obesity across childhood and adolescence in a large, contemporary, cohort of English children. A secondary aim was to examine the persistence of overweight and obesity.

## Methods

### Study cohort

ALSPAC (The Avon Longitudinal Study of Parents and Children) is a large prospective cohort study of children born in the South-West of England in 1991/1992; study design and methods are described elsewhere (Ness, 2004, Golding and the ALSPAC Study Team, 1996). Briefly, 14,541 pregnant women with an expected date of delivery between April 1991 and December 1992 were enrolled, resulting in 13,988 participating children alive at one year. Detailed information has been collected using self-administered questionnaires, data extraction from medical notes, linkage to routine information systems and at research clinics for children. A 10% sample of the ALSPAC cohort, the Children in Focus (CiF) group, attended research clinics at 4, 8, 12, 18, 25, 31, 37, 43, 49, and 61 months where detailed physical examinations were undertaken. The CiF group was broadly socio-economically representative of the entire ALSPAC cohort and the UK (Reilly et al., 2005). From age 7, the entire ALSPAC cohort was invited to attend regular research clinics. Ethical approval for the study was obtained from the ALSPAC Law and Ethics Committee and local Health Service Research Ethics Committees.

### Study procedures

At each measurement occasion, height was measured to 0.1 cm and weight was measured to 0.1 kg in underwear. BMI was calculated as weight (kg) / length (m)^2^. Weight status was defined using BMI z-scores relative to UK 1990 BMI population reference data: healthy weight (BMI z-score < 1.04, below the 85th percentile); overweight (BMI z-score ≥ 1.04–< 1.64, equivalent to 85th–94th percentiles); obese (BMI z-score ≥ 1.64, equivalent to ≥ 95th percentile). These definitions have high specificity and high sensitivity for the identification of children with high fat mass, and diagnostic accuracy does not differ significantly between the sexes (Reilly et al., 2000, Reilly et al., 2010). The International Obesity Task Force definitions of overweight and obesity were not used in the present study because they have much lower sensitivity than definitions based on UK reference data in UK children, and have marked differences in sensitivity between the sexes (Reilly et al., 2000, Reilly et al., 2010).

We addressed the aims of the present study using the ALSPAC CiF subsample (with measures made annually from age 3 years) because this provided data across childhood and adolescence. As a check, we also used the entire ALSPAC cohort because the sample size is much larger, though annual BMI measurements were available for the entire sample only from age 7 to 15 years.

### Statistical analysis

Due to high prevalence of overweight and obesity (> 20%) at all ages, risk ratios for overweight and obesity at 15 years based on weight status at 3, 7 and 11 years were calculated.

We re-ran all analyses (for the CiF sample and the entire ALSPAC cohort) restricting the analyses to participants with data at all time periods (n = 521 for CiF group and n = 4283 for entire ALSPAC cohort) and similar results were obtained. We compared study participants with data at 3, 7 and 15 years (n = 549) to those with data at 3 and 7 years but not 15 years (n = 288) for the CiF subsample for a number of characteristics using independent sample t-tests/chi squared tests: 95% confidence intervals for the differences are presented along with p-values. We also compared study participants with data at 7, 11 and 15 years (n = 4283) to those with data at 7 and 11 years but not 15 years (n = 1626) for the entire ALSPAC cohort for a number of characteristics using independent sample t tests t-tests/chi squared tests.

## Results

### Characteristics of study participants and influence of loss to follow up

Characteristics of study participants who were followed up and those lost to follow up are shown in Table 1 for the *CiF sample* and Table 2 for the *entire ALSPAC cohort*. We compared study participants with data at 3, 7 and 15 years (n = 549) to those with data at 3 and 7 years but not 15 years (n = 288) for the *CiF sample*. Slightly more boys were lost to follow-up, however parental obesity, markers of socio-economic position, and BMI z-scores were similar between those followed up and lost to follow up (Table 1). We compared study participants with data at 7, 11 and 15 years (n = 4283) to those with data at 7 and 11 years but not 15 years (n = 1626) for the *entire ALSPAC cohort*. Slightly more boys were lost to follow-up, those lost to follow up had lower SES, higher BMI z-score at 11 years and higher parental obesity than those followed up (see Table 2), though differences were small.

**Table 1:** Characteristics of study participants followed-up (n = 549) and those not followed-up (n = 288) in the Children in Focus (CiF) sample, England, 1991–2007.

	Followed up		Not followed up		P for difference
	n	Mean (SD) or n (%)	n	Mean (SD) or n (%)	
Gender	549		288		0.003
Boys		276 (50.3)		176 (61.1)	
Girls		273 (49.7)		112 (38.9)	
Mother's education	546		278		0.31
School to age 16 years		286 (52.4)		156 (56.1)	
Education to 18 years or beyond		260 (47.6)		122 (43.9)	
Mother obese	514	27 (5.3)	259	17 (6.6)	0.46
Partner obese	444	37 (8.3)	191	13 (6.8)	0.51
BMI z score at 3 years	549	0.33 (0.94)	288	0.21 (0.98)	0.10
BMI z score at 7 years	549	0.18 (1.07)	288	0.07 (1.03)	0.15
BMI z score at 11 years	521	0.38 (1.17)	176	0.42 (1.23)	0.69

Parental obesity defined as BMI > 30 based on self reported weights and heights at 12 weeks gestation.

**Table 2:** Characteristics of study participants followed-up (n = 4283) and those not followed-up (n = 1626) in the entire ALSPAC cohort, England, 1991–2007.

	Followed up		Not followed up		P for difference
	n	Mean (SD) or n (%)	n	Mean (SD) or n (%)	
Gender	4283		1626		0.001
Boys		2070 (48.3)		873 (53.7)	
Girls		2213 (51.7)		753 (46.3)	
Mother's education	4190		1553		0.001
School to age 16 years		2161 (51.6)		1005 (64.7)	
Education to 18 years or beyond		2029 (48.4)		548 (35.3)	
Mother obese	3948	185 (4.7)	1455	89 (6.1)	0.03
Partner obese	3210	227 (7.1)	1060	94 (8.9)	0.05
BMI z score at 7 years	4283	0.10 (1.02)	1626	0.12 (1.09)	0.49
BMI z score at 11 years	4283	0.32 (1.16)	1626	0.39 (1.24)	0.04

Parental obesity defined as BMI > 30 based on self reported weights and heights at 12 weeks gestation.

Prevalence of healthy weight (BMI < 85th centile), overweight (BMI 85th–94th centile) and obesity (BMI at or above 95th centile) are described in Table 3 for the *CiF sample* and Table 4 for the *entire cohort*. Prevalence of overweight and obesity was similar between the CIF sample and the entire ALSPAC cohort and between boys and girls.

**Table 3:** Prevalence of healthy weight, overweight and obesity at each time period in the Children in Focus (CiF) sample, England, 1991–2007.

	3 year clinic	7 year clinic	11 year clinic	15 year clinic
	n = 1051^a^	n = 964^a^	n = 864^a^	n = 651^a^
Mean age, months (SD)	37.0 (0.2)	89.3 (1.6)	141.2 (2.6)	184.4 (2.3)
(Range)	(36–38)	(82–98)	(128–150)	(173–196)
Median BMI (IQR)	16.4 (15.6–17.2)	15.8 (14.9–17.0)	18.4 (16.7–20.9)	20.9 (19.1–23.0)
Mean BMI z score (SD)	0.31 (0.96)	0.14 (1.06)	0.41 (1.19)	0.41 (1.14)
Healthy weight % (n)	80.5 (846)	82.5 (795)	69.1 (597)	74.3 (484)
Overweight % (n)	11.6 (122)	8.8 (85)	14.5 (125)	12.3 (80)
Obese % (n)	7.9 (83)	8.7 (84)	16.4 (142)	13.4 (87)
Overweight and obese % (n)	19.5 (205)	17.5 (169)	30.9 (267)	25.7 (167)

Number attending clinic; n = 1081 at 3 years, n = 978 at 7 years, n = 868 at 11 years, n = 662 at 15 years.

Abbreviations: IQR: inter-quartile range; SD: standard deviation.

Healthy weight: below 85th centile for BMI. Overweight: 85th–94th centiles for BMI; obese ≥ 95th centile for BMI.

a Number of individuals providing data to calculate BMI.

**Table 4:** Prevalence of healthy weight, overweight and obesity among participants at each time period in the entire ALSPAC cohort, England, 1991–2007.

	All	Boys	Girls
7 year clinic (n)^a^	7759	3946	3813
Mean age in months (SD)	89.9 (2.3)	89.8 (2.3)	89.8 (2.3)
(Range)	(82–110)	(82–109)	(84–110)
Median BMI (IQR)	15.8 (14.9–17.1)	15.7 (14.8–16.8)	15.9 (14.9–17.3)
Mean BMI z score (SD)	0.13 (1.04)	0.12 (1.05)	0.13 (1.02)
Healthy weight % (n)	82.4 (6394)	82.9 (3273)	81.9 (3121)
Overweight % (n)	9.2 (714)	8.3 (328)	10.1 (386)
Obese % (n)	8.4 (651)	8.7 (345)	8.0 (306)
Overweight and obese % (n)	17.6 (1365)	17.0 (673)	18.1 (692)

11 year clinic (n)^a^	6751	3341	3410

Mean age in months (SD)	141.0 (2.9)	140.9 (2.8)	141.0 (2.9)
(Range)	(125–163)	(128–162)	(125–163)
Median BMI (IQR)	18.3 (16.6–20.8)	18.0 (16.5–20.5)	18.6 (16.8–21.2)
Mean BMI z score (SD)	0.36 (1.19)	0.40 (1.19)	0.31 (1.19)
Healthy weight % (n)	70.8 (4778)	69.8 (2332)	71.7 (2446)
Overweight % (n)	13.4 (907)	13.4 (447)	13.5 (460)
Obese % (n)	15.8 (1066)	16.8 (562)	14.8 (504)
Overweight and obese % (n)	29.2 (1973)	30.2 (1009)	28.3 (964)

15 year clinic (n)^a^	5162	2449	2713

Mean age in months (SD)	185.7 (4.2)	185.4 (3.9)	185.9 (4.5)
(Range)	(171, 212)	(174, 212)	(171, 210)
Median BMI (IQR)	20.7 (19.0, 23.0)	20.3 (18.8, 22.4)	21.1 (19.3, 23.4)
Mean z score (SD)	0.36 (1.09)	0.35 (1.08)	0.38 (1.10)
Healthy weight % (n)	74.7 (3855)	75.0 (1837)	74.4 (2018)
Overweight % (n)	12.9 (666)	12.8 (313)	13.0 (353)
Obese % (n)	12.4 (641)	12.2 (299)	12.6 (342)
Overweight and obese % (n)	25.3 (1307)	25.0 (612)	25.6 (695)

Number attending clinic; n = 7834 at 7 years, n = 6794 at 11 years, n = 5247 at 15 years.

IQR = Inter-quartile range.

Healthy weight: BMI < 85th centile. Overweight: BMI 85th–94th centile; obese: BMI ≥ 95th centile.

a Number of individuals providing data to calculate BMI.

### Incidence of overweight and obesity throughout childhood and adolescence in the CiF sample

There was some differential loss to follow up from 3 to 7 years and 11 to 15 years. Children who were obese at 3 years were slightly more likely to be lost to follow-up at 7 years [25.3% (21/83)] than those overweight at 3 years [23.0% (28/122)] or healthy weight at 3 years [19.5% (165/846)]. Children who were obese at 11 years were slightly more likely to be lost to follow up at 15 years [35.2% (50/142)] than children overweight [31.2% (39/125)] or healthy weight [28.5% (170/597)]. From 7 to 11 years, loss to follow up was similar in each group (~ 18%).

The incidence of overweight and obesity in the CiF sample from 3 to 7, 7 to 11, and 11 to 15 years is shown in Table 5A. Incidence of overweight and obesity was higher from 7 to 11 years [overweight 11.8% (76/646); obese 6.7% (43/646)] than 3 to 7 years [overweight 5.3% (36/681); obese 5.1% (35/681)] and 11 to 15 years [overweight 5.6% (24/427); obese 1.6% (7/427)].

**Table 5:** Four year incidence of overweight and obesity n, (%) in the Children in Focus (CiF) sub-sample from age 3 to 15 years (5A) and in the entire ALSPAC cohort (5B) from age 7 to 15 years, England, 1991–2007.

5A Children in Focus (CiF) subsample			
3–7 years	Healthy weight at 3 years	Overweight at 7 years	Obese at 7 years
	681	36 (5.3)	35 (5.1)
7–11 years	Healthy weight at 7 years	Overweight at 11 years	Obese at 11 years
	646	76 (11.8)	43 (6.7)
11–15 years	Healthy weight at 11 years	Overweight at 15 years	Obese at 15 years
	427	24 (5.6)	7 (1.6)

5B Entire ALSPAC cohort			

7–11 years	Healthy weight at 7 years	Overweight at 11 years	Obese at 11 years
	4895	588 (11.4)	243 (5.0)
11–15 years	Healthy weight at 11 years	Overweight at 15 years	Obese at 15 years
	3361	220 (6.5)	47 (1.4)

### Incidence of overweight and obesity throughout childhood and adolescence in the entire ALSPAC cohort

There was some differential loss to follow up from 7 to 11 years and 11 to 15 years. Children obese at 7 years were slightly more likely to be lost to follow up at 11 years [28.3% (184/651)] than those overweight at 7 [23.4% (167/714)] or healthy weight at 7 [23.4% (1499/6394)]. Children obese at 11 years were slightly more likely to be lost to follow up at 15 [35.3% (376/1066)] than children who were overweight [32.1% (291/907)] or healthy weight [29.7% (1417/4778)].

Incidence of overweight [11.4% (558/4895)] and obesity [5.0% (243/4895)] from 7 to 11 years in the *entire cohort* was higher than the incidence from 11 to 15 years [overweight 6.5% (220/3361); obese 1.4% (47/3361)], see Table 5B. In addition, the incidence of overweight was slightly higher than the incidence of obesity at each time period. Furthermore, incidence of overweight and obesity from 7 to 11 years and from 11 to 15 years was similar between boys and girls and to the entire ALSPAC cohort (for full results see Supplementary Web Figs. 1 and 2 in Appendix A).

### Risk ratios for overweight or obese at age 15 years based on weight status at 3, 7 and 11 years in the CiF sample

In the CiF sample, 47.3% (52/110) of children who were overweight and obese at 3 years were overweight and obese at 15 years compared to 20% (93/465) of children who were healthy weight at 3 years; 70% (77/110) of children who were overweight and obese at 7 years were overweight and obese at 15 years compared to 15.3% (75/491) of children who were healthy weight at 7 years; 67.4% (120/178) of children who were overweight and obese at 11 years were overweight and obese at 15 years compared to 7.3% (31/427) of children who were healthy weight at 11 years.

The risk ratios for overweight and obesity at 15 years from overweight and obesity at 3, 7 and 11 years (relative to healthy weight status at 3, 7 and 11 years) are shown in Table 6. Children who were overweight or obese at age 3, 7 or 11 years were at much greater risk of being overweight or obese at age 15 years relative to healthy weight children at each time point. In addition, the risk of a child being overweight or obese at 15 years was much higher if they were overweight or obese at 11 years compared to being overweight or obese at 3 and 7 years.

**Table 6:** Risk ratios for overweight and obesity at 15 years from overweight and obesity at 3, 7 and 11 years (relative to healthy weight status at 3, 7, and 11 years), England, 1991–2007.

	CiF group		Entire ALSPAC cohort	
	n	Risk ratios (95% CI)	n	Risk ratios (95% CI)
3 to 15 years	575	2.4 (1.8, 3.1)	Not applicable	Not applicable
7 to 15 years	601	4.6 (3.6, 5.8)	4572	5.1 (4.7, 5.6)
11 to 15 years	605	9.3 (6.5, 13.2)	4667	8.6 (7.6, 9.7)

Risk ratios and sample sizes in entire ALSPAC cohort not applicable from ages 3–15 years because data only collected on entire cohort from age 7 years.

### Risk ratios for overweight or obese at age 15 years based on weight status at 7 and 11 years in the entire ALSPAC cohort

In the entire ALSPAC cohort, 73.7% (569/772) of children who were overweight and obese at 7 years were overweight and obese at 15 years compared to 14.5% (550/3800) of children who were healthy weight at 7 years; 68.2% (891/1306) of children who were overweight and obese at 11 years were overweight and obese at 15 years compared to 7.9% (267/3361) of children who were healthy weight at 11 years.

The risk ratios for overweight and obesity at 15 years from overweight and obesity at 7 and 11 years (relative to healthy weight status at 7 and 11 years) *for the entire cohort* are shown in Table 6. Children who were overweight or obese at age 7 or 11 years were at much greater risk of being overweight or obese at age 15 years relative to healthy weight children at each time point. In addition, the risk of a child being overweight or obese at 15 years was much higher if they were overweight or obese at 11 years compared to being overweight or obese at age 7 years.

## Discussion

In the present study incidence of overweight and obesity varied markedly by age, with peak incidence in mid–late childhood (age 7–11 years). Previous obesity prevention interventions have often had limited impact (Summerbell et al., 2005, Kamath et al., 2008): one possibility is that such interventions do not take sufficient account of the ‘background’ incidence of obesity in the populations under study. While the tendency of overweight and obesity to persist is established, quantitative estimates of persistence from large contemporary cohorts which have used modern, accepted, overweight and obesity definitions are rare (Reilly et al., in press, Singh et al., 2008): such estimates could inform future prevention strategies. It should also be noted that overweight and obesity during childhood and obesity can resolve (Reilly et al., in press).

### Comparisons with other evidence

The only directly comparable UK study is that of Wardle et al. (2006), which found that incidence of obesity was low between ages 11 and 15 years, consistent with the results of the present study. In a previous study of the ALSPAC cohort we examined the timing of excess weight gain across the entire distribution of weight status (Hughes et al., 2011): this suggested that mid–late childhood is the period when contemporary English children are most prone to excessive weight gain, consistent with the suggestion from the present study that mid–late childhood should be a focus for future preventive interventions. Reilly et al. (in press) examined the probability of progression to from overweight to obesity in ALSPAC, but only from ages 7 to 13 years.

The differences in obesity incidence by age found in the present study might reflect differences in lifestyle at different ages which alter susceptibility to obesity, or differences in the extent to which the environment promoted obesity at different times—a period effect. However, given the short period of time over which the present study took place, and the steady progression of the obesity epidemic in English children during the 1990s (Reilly and Dorosty, 1999, Stamatakis et al., 2010), the present study suggests that mid–late childhood in England may be particularly ‘obesogenic’.

### Study strengths and limitations

The present study had a number of strengths: longitudinal design; large sample size; contemporary and broadly socio-economically representative nature of the cohort; wide age span of the cohort across childhood and adolescence. One weakness of the present study may be generalisability. A degree of attrition in longitudinal studies is inevitable. We provided analyses which help interpret the possible impact of attrition, and some characteristics of participants lost to follow up differed slightly from those retained to older ages, including a tendency for higher BMI z score in those lost to follow up. The present study did not use the International Obesity Task Force definition of child and adolescent obesity because the low sensitivity of this definition (Reilly et al., 2000) produced very small numbers of incident cases of obesity, reducing power. In addition, the substantial differences in sensitivity between the sexes when the International Obesity Task Force definition was used limited the ability to combine incidence data from both sexes.

## Conclusions

Development of overweight and obesity is greatest during mid–late childhood in the UK. Future interventions to prevent child and adolescent obesity might consider greater targeting of obesity prevention in mid–late childhood (age 7–11 years).

## Conflict of interest statement

The authors declare that there are no conflicts of interest.
