# Supplementary material for: Incidence of obesity during childhood and adolescence in a large contemporary cohort
Source: Prev Med. 2011 May 1;52(5):300–4. doi: 10.1016/j.ypmed.2011.02.014 (PMC3919170; doi:10.1016/j.ypmed.2011.02.014)
Supplement: Supplementary file 1 — Supplementary materials. [file mmc1.pdf]

APPENDIX 1 –Additional materials to be considered as web/supplementary tables

Incidence of overweight and obesity throughout childhood in the entire ALSPAC cohort among boys and girls , England, 1991-2007

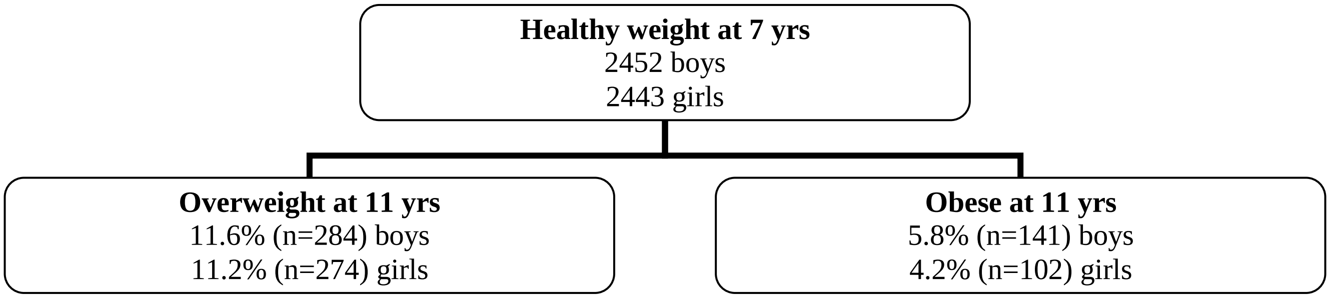

Figure 1: Incidence of overweight and obesity among boys and girls from 7 years to 11 years in the entire ALSPAC cohort. Only those who were followed up at 11 years were used to calculate the incidence of overweight and obesity.

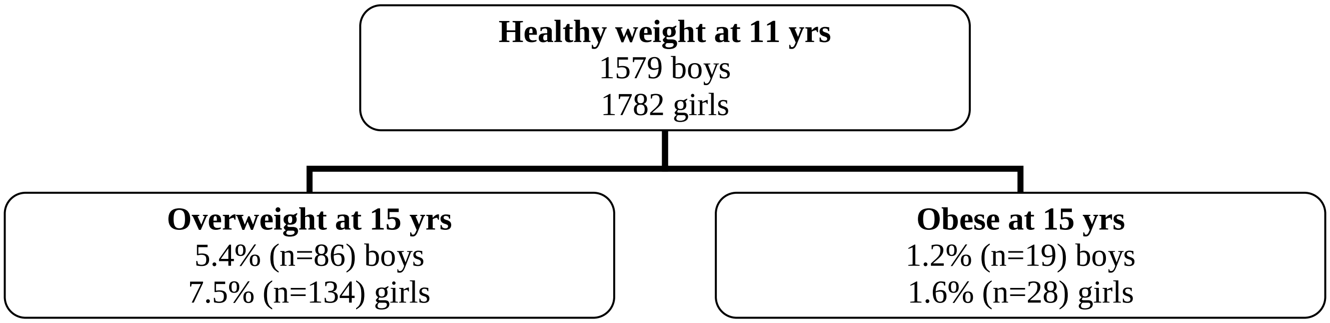

Figure 2: Incidence of overweight and obesity among boys and girls from 11 to 15 years in the entire ALSPAC cohort. Only those who were followed up at 15 years were used to calculate the incidence of overweight and obesity.

CIF SAMPLE

Table 1: Children in each weight status category at age 7 by weight status category at age 3 years in the CiF group, n (%), England, 1991-2007

	Healthy weight at 7 yrs n=795	Overweight at 7 yrs n=85	Obese at 7 yrs n=84	Missing at 7 yrs but not at 3 yrs
**Healthy weight at 3 yrs n=846**	610 (72.1)	36 (4.2)	35 (4.1)	165 (19.5)
**Overweight at 3 yrs n=122**	60 (49.2)	19 (15.6)	15 (12.3)	28 (23.0)
**Obese at 3 yrs n=83**	22 (26.5)	17 (20.5)	23 (27.7)	21 (25.3)
**Missing at 3 yrs but not at 7 yrs**	103 (81.1)	13 (10.2)	11 (8.7)	

Footnote: used the total number of healthy weight children at 3 years (n=846) to calculate the % of healthy weight, overweight and obese children at 7 years as well as the % missing at 7 years. Footnote:
The missing categories include those not attending the measurement clinic and those attending the clinic but not providing data to calculate BMI.

Table 2: Proportion of children in each weight status category at age 11 by weight status category at age 7 years in the CiF group, n (%), England , 1991-2007

	Healthy weight at 11 yrs n=597	Overweight at 11 yrs n=125	Obese at 11 yrs n=142	Missing at 11 yrs but not 7 yrs
**Healthy weight at 7 yrs n=795**	527 (66.3)	76 (9.6)	43 (5.4)	149 (18.7)
**Overweight at 7 yrs n=85**	14 (16.5)	28 (32.9)	28 (32.9)	15 (17.6)
**Obese at 7 yrs n=84**	6 (7.1)	6 (7.1)	57 (67.8)	15 (17.9)
**Missing at 7 yrs but not at 11 yrs**	50 (63.3)	15 (19.0)	14 (17.7)	

Table 3: Proportion of children in each weight status category at age 15 by weight status category at age 11 years in the CIF group, n (%), England, 1991-2007

	Healthy weight at 15 yrs n=484	Overweight at 15 yrs n=80	Obese at 15 yrs n=87	Missing at 15 yrs but not at 11 yrs
**Healthy weight at 11 yrs n=597**	396 (66.3)	24 (4.0)	7 (1.2)	170 (28.5)
**Overweight at 11 yrs n=125**	40 (32.0)	32 (25.6)	14 (11.2)	39 (31.2)
**Obese at 11 yrs n=142**	18 (12.7)	19 (13.4)	55 (38.7)	50 (35.2)
**Missing at 11 yrs but not at 15 yrs**	30 (65.2)	5 (10.9)	11 (23.9)	

ENTIRE ALSPAC COHORT

Table 4: Proportion of children in each weight status category at age 11 by weight status category at age 7 years in the entire ALSPAC cohort, n (%), England, 1991-2007

	Healthy weight at 11 yrs n=4778	Overweight at 11 yrs n=907	Obese at 11 yrs n=1066	Missing at 11 yrs but not at 7 yrs
**Healthy weight at 7 yrs n=6394**	4094 (64.0)	558 (8.7)	243 (3.8)	1499 (23.4)
**Overweight at 7 yrs n=714**	104 (14.5)	194 (27.2)	249 (34.9)	167 (23.4)
**Obese at 7 yrs n=651**	17 (2.6)	43 (6.6)	407 (62.5)	184 (28.3)
**Missing at 7 yrs but not 11 yrs**	563 (66.9)	112 (13.3)	167 (19.8)	

Table 5: Proportion of children in each weight status category at age 15 by weight status category at age 11 years in the entire ALSPAC cohort, n (%), England, 1991-2007

	Healthy weight at 15 yrs n=3855	Overweight at 15 yrs n=666	Obese at 15 yrs n=641	Missing at 15 yrs but not at 11 yrs
**Healthy weight at 11 yrs n=4778**	3094 (64.8)	220 (4.6)	47 (1.0)	1417 (29.7)
**Overweight at 11 yrs n=907**	304 (33.5)	206 (22.7)	106 (11.7)	291 (32.1)
**Obese at 11 yrs n=1066**	111 (10.4)	170 (15.9)	409 (38.4)	376 (35.3)
**Missing at 11 yrs but not at 15 yrs**	346 (69.9)	70 (14.1)	79 (16.0)	

BOYS AND GIRLS (ENTIRE ALSPAC COHORT)

The proportion of boys and girls in each weight status category at age 11 by weight status category at age 7 years is shown in Table 5a and 5b.

Table 5a: Proportion of BOYS in each weight status category at age 11 by weight status category at age 7 years in the entire ALSPAC cohort, n (%), England, 1991-2007

	Healthy weight at 11 yrs n=2332	Overweight at 11 yrs n=447	Obese at 11 yrs n=562	Missing at 11 yrs but not at 7 yrs
**Healthy weight at 7 yrs n=3273**	2027 (61.9)	284 (8.7)	141 (4.3)	821 (25.1)
**Overweight at 7 yrs n=328**	40 (12.2)	92 (28.0)	113 (34.4)	83 (25.3)
**Obese at 7 yrs n=345**	7 (2.0)	16 (4.6)	223 (64.6)	99 (28.7)
**Missing at 7 yrs but not 11 yrs**	258 (64.8)	55 (13.8)	85 (21.4)	

Table 5b: Proportion of GIRLS in each weight status category at age 11 by weight status category at age 7 years in the entire ALSPAC cohort, n (%), England, 1991-2007

	Healthy weight at 11 yrs n=2446	Overweight at 11 yrs n=460	Obese at 11 yrs n=504	Missing at 11 yrs but not at 7 yrs
**Healthy weight at 7 yrs n=3121**	2067 (66.2)	274 (8.8)	102 (3.3)	678 (21.7)
**Overweight at 7 yrs n=386**	64 (16.6)	102 (26.4)	136 (35.2)	84 (21.8)
**Obese at 7 yrs n=306**	10 (3.3)	27 (8.8)	184 (60.1)	85 (27.8)
**Missing at 7 years but not 11 yrs**	305 (68.7)	57 (12.8)	82 (18.5)	

The proportion of boys and girls in each weight status category at age 15 by weight status category at age 11 years is shown in Table 6a and 6b.

Table 6a: Proportion of boys in each weight status category at age 15 by weight status category at age 11 years in the entire ALSPAC cohort, n (%), England 1991-2007

	Healthy weight at 15 yrs n=1837	Overweight at 15 yrs n=313	Obese at 15 yrs n=299	Missing at 15 yrs but not at 11 yrs
**Healthy weight at 11 yrs n=2332**	1474 (63.2)	86 (3.7)	19 (0.8)	753 (32.3)
**Overweight at 11 yrs n=447**	163 (36.5)	102 (22.8)	38 (8.5)	144 (32.2)
**Obese at 11 yrs n=562**	60 (10.7)	90 (16.0)	213 (37.9)	199 (35.4)
**Missing at 11 yrs but not at 15 yrs**	140 (26.9)	351 (67.5)	29 (5.6)	

Table 6b: Proportion of Girls in each weight status category at age 15 by weight status category at age 11 years in the entire ALSPAC cohort, England, n (%), 1991-2007

	Healthy weight at 15 yrs n=2018	Overweight at 15 yrs n=353	Obese at 15 yrs n=342	Missing at 15 yrs but not at 11 yrs
**Healthy weight at 11 yrs n=2446**	1620 (66.2)	134 (5.5)	28 (1.1)	664 (27.1)
**Overweight at 11 yrs n=460**	141 (30.6)	104 (22.6)	68 (14.8)	147 (32.0)
**Obese at 11 yrs n=504**	51 (10.1)	80 (15.9)	196 (38.9)	177 (35.1)
**Missing at 11 yrs but not at 15 yrs**	206 (70.8)	35 (12.0)	50 (17.2)	
